# Patients with Clinically Suspected but Unproven Hip Fractures, Who Require Cross-Sectional Imaging, Are Best Initially Admitted under Geriatrician-Led Care—A Retrospective Review

**DOI:** 10.3390/geriatrics3040068

**Published:** 2018-10-10

**Authors:** Hamish Macdonald, Niraj Vetharajan, Peter Kempshall

**Affiliations:** Gloucestershire Hospitals NHS Foundation Trust, Cheltenham, GL53 7AN, UK; hamish.macdonald@doctors.org.uk (H.M.); nirajvetharajan@gmail.com (N.V.)

**Keywords:** hip fracture, neck of femur, proximal femoral fracture, admissions, orthogeriatrics, orthopaedics

## Abstract

Patients with suspected hip fractures who require further imaging to confirm or disprove the diagnosis may be admitted to orthopaedic or medical departments. We aim to provide evidence regarding the appropriate admission pathway for such patients. This is a retrospective study of all suspected hip fracture patients receiving second-line imaging between 1 January 2015 to 30 June 2016 in one hospital trust. Information was gained from hospital records to determine indication and result of imaging, eventual diagnoses, length of stay, and inpatient mortality. During the study period, 126 patients underwent cross-sectional imaging for clinically suspected but unproven hip fractures. Of these, 27% were positive for hip fractures (*n* = 34, 3.2% of hip fracture admissions) whilst the remainder were negative. Of the patients without hip fractures, 50 (54%) had a concomitant medical discharge diagnosis. Thirty-one different diagnoses were found in this cohort. This research provides evidence for geriatrician-led admission of patients with suspected but unproven hip fracture, due to the frailty and medical requirements of this patient group.

## 1. Introduction

In recent years, the management of elderly patients with hip fractures in the United Kingdom has developed, with demonstrable improvements in morbidity and mortality [1]. It is recognised that such patients are a vulnerable group who suffer multiple medical co-morbidities, which impact on morbidity and mortality [2,3].

National targets require joint orthopaedic and medical orthogeriatric care for best outcomes. The 30-day mortality in this group is 6.7% nationally [4]. We hypothesized that patients with a clinically suspected hip fracture, but with a normal or equivocal plain radiograph, may fall into the same high-risk comorbid population.

Much research has been performed into the best diagnostic pathways when patients present with a clinical hip fracture but initial radiographs are negative or equivocal [5,6] Guidelines have been formulated [7] stating that the optimum form of investigation is an urgent magnetic resonance imaging (MRI) scan, or a computed tomography (CT) scan when the former is not available or is clinically inappropriate. In current literature, when cross-sectional imaging is performed for suspected hip fracture, it is positive in 30% of patients [4,5].

However, little has been published on the outcomes of the remaining patients who do not have a hip fracture. Here we present an analysis of all patients who underwent cross-sectional imaging (MRI and/or CT scanning) in one trust in an 18-month period for suspected hip fracture, to determine diagnoses and eventual outcomes.

As hospitals regularly reach 105% of their operating capacity, increasing attention has been placed on streamlining admission pathways. All hospital departments are under pressure to reduce admission numbers to alleviate pressures on their service. Getting patients to the “right place, first time” provides best patient care, improves efficiency, is less demanding on resources, and reduces length of stay.

This is the first article that aims to provide an evidence base for appropriate admission of patients with suspected but unproven hip fracture.

## 2. Materials and Methods

From a single NHS foundation trust, consisting of two district general hospitals both receiving hip fracture patients, all second-line imaging (MRI and CT of pelvis or hips) was reviewed for an 18-month period between 1 January 2015 and 30 June 2016. The policy in our trust was for such patients to be admitted under the medical team and to receive a physiotherapy assessment and analgesia. If they remained unable to mobilise without significant pain, then they underwent cross-sectional imaging.

Patients were only included if aged 65 and over. All second-line imaging performed with the aim of identifying or excluding hip fractures was reviewed. The scan reports and National Hip Fracture Database (NHFD) were cross-referenced to identify which patients had hip fractures, and whether these had been identified by second-line imaging.

Discharge summaries and medical notes were reviewed to identify the following data: the admitting team, whether the patient died during the admission, the presence or absence of other pelvic/hip injury (specifically fractures of the pubic ramus, sacrum, acetabulum or greater trochanter), length of stay, and discharge diagnosis (causes of death for deceased patients).

As a retrospective notes-based review, with no impact on the care of the patients included in this study, trust ethics board approval was waived.

## 3. Results

### 3.1. Overview and Demographics

One hundred and twenty-six second-line imaging studies were performed for suspected occult fractured hips in our institutions during the study period. In the same time frame, 1047 patients were admitted with fractured hips. Of the 126 second-line imaging studies performed, 34 were positive for hip fractures (27%) whilst the remaining 92 (73%) did not have such injuries. The demographic data of the cohort is shown in Table 1.

### 3.2. Investigations Performed

**Figure 1:**
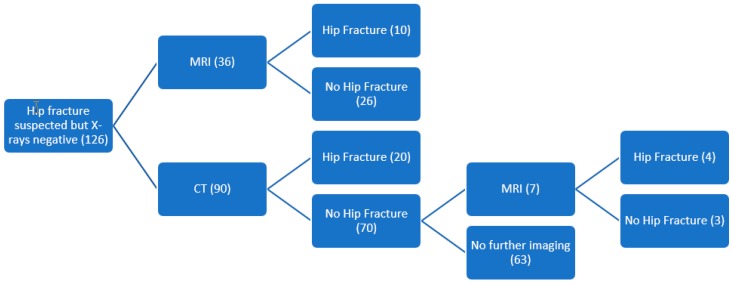
Cross-sectional imaging performed and its outcomes.

Of the 126 scans performed for suspected occult hip fracture, MRI was performed in the first instance for 36 patients and CT for the remaining 90. Ten patients who had MRI (28%) were proven to have a hip fracture, whilst 26 (72%) were negative for hip fractures. Of the 90 patients who underwent CT scanning, 20 (22%) were proven to have a hip fracture, whilst the remaining 70 patients (78%) had a negative scan. Of the negative scans, seven patients (10%) had further imaging in the form of MRI, which was positive in four cases (57%). The investigations and their results are summarized in Figure 1.

### 3.3. Admission Pathway

In our trust, the policy is for patients with a suspected but unproven hip fracture to be admitted under medical-led teams rather than orthopaedics, and this policy was followed in the majority of cases. Of the 126 patients undergoing cross-sectional imaging, 22 (17%) were initially admitted under orthopaedics, whilst 104 (83%) were admitted under medical teams. Patients who subsequently proved to have a neck of femur fracture were then transferred to shared orthopaedic and orthogeriatric care, but the majority (32, 94%) were initially admitted under medical physician. The majority of patients (92, 73%) did not have a hip fracture. Of this cohort, 20 (22%) were admitted under orthopaedics whilst awaiting their scan, in contradiction of trust policy, with the remaining 78% admitted under medical teams.

### 3.4. Discharge Diagnoses of Patients Who Did Not Have a Hip Fracture

#### 3.4.1. Medical Diagnoses

In the non-hip fracture cohort, 50 patients (54%) had significant medical diagnoses listed on their discharge paperwork. In total, there were 31 different medical diagnoses listed, with a cumulative total of 69 listed diagnoses. The most common medical discharge diagnosis was urinary tract infection including urosepsis (14 occasions, 15%), followed by hospital-acquired pneumonia (nine occasions, 10%) and community-acquired pneumonia (seven occasions, 8%). A complete indication of the discharge diagnoses of all patients is shown in Table 2.

#### 3.4.2. Orthopaedic Diagnoses

Twenty-eight (30%) of the patients without hip fractures had another fragility fracture of the pelvis. Of these the majority (19, 15% of all patients scanned) had pubic rami fractures, and less frequently, fractures of the sacral alar (11, 9%), acetabulum (7, 6%), and greater trochanter (3, 2%). No patient undergoing second-line imaging for suspected hip fracture, who did not have a proven hip fracture, required inpatient orthopaedic intervention.

#### 3.4.3. Mortality and Causes of Death

Three patients (3%) died as inpatients within the cohort of patients without hip fracture. None of the small group of patients with a hip fracture died before discharge, although one was discharged without an operation back to her nursing home for end-of-life care. The causes of death for the patients who died in hospital are listed below (1a refers to the direct cause if death, 1b to any contributing factors to 1a, and 2 to any additional relevant diagnoses that have not directly led to 1a; these subheadings are those used on United Kingdom death certificates):

- 1a Bronchopneumonia
- 1a Carcinomatosis, 1b Gastric cancer, bronchial cancer
- 1a Frailty of old age, 2 Advanced dementia

## 4. Discussion

There is considerable inter-hospital variability in the admitting pathway and admission location of these patients both before and after their second-line imaging for suspected hip fractures. The authors believe that the ideal admission pathway would be via a “frailty unit” that has shared orthopaedic and geriatric care, with access to the same rehabilitative facilities such as exercise classes, cognitive therapy sessions, a communal eating program, and enhanced nutrition, as is currently available in our hip fracture unit. Limiting this pathway however, is that many units cannot physically staff a designated “frailty unit” due to shortages of care of the elderly medical staff and pressure on state facilities.

Due to high demand for the cross-sectional imaging, radiology departments are often unable to provide this service within the four-hour time limit imposed on emergency departments and so must be admitted under a specialist team. Some centres admit these patients under the medical or rehabilitation teams, and others under orthopaedics.

In this paper, we have shown that over half (54%) of these patients either have on admission or develop a significant medical problem during their admission. The scope of these diagnoses is broad and requires expert input in order that these patients have the best chance of a favourable outcome.

Although the sample size is small, it should be noted that the inpatient mortality rate in patients who did not have a hip fracture was 3.3%. While this figure is lower than the national 30-day figure of 6.7% [4] for patients who have hip fractures, it underlines that there is an ever-present mortality rate among this group of elderly patients who happen to present following a minor fall. In addition, as the average length of stay was less than 30 days, there is a chance that the true 30-day mortality is greater than this figure.

As a retrospective piece of work, the study is reliant on the accuracy of the hospital discharge summary which is a recognised limitation. [8,9] As such it seems likely that there were additional diagnoses that we were not able to include in this analysis which would further support the admission pathway.

Regarding the choice of imaging modality, this work supports the conclusion of the National Institute for Health and Care Excellence guidelines on hip fracture care [4] which state: “Offer magnetic resonance imaging (MRI) if hip fracture is suspected despite negative X-rays of the hip of an adequate standard. If MRI is not available within 24 h or is contraindicated, consider computed tomography (CT)”. Even with modern CT scanning, in this study 54% of patients who had an MRI following a negative CT were shown to have a hip fracture. It should be noted that MRI scans were only performed following a negative CT scan if there was ongoing clinical suspicion (i.e., inability to weight bear despite analgesia and physiotherapy assessment). It should also be noted that none of the patients with a negative CT scan, who did not undergo subsequent MRI scanning, later presented with a fracture. From this we conclude that in the context of a negative CT scan, patients do not necessarily require an MRI scan if their clinical picture improves such that they are able to bear weight. If however patients remain unable to bear weight, then this study shows that CT is unable to definitively rule out a fracture and an urgent MRI should be performed. Urgent MRI remains the investigation of choice to identify an occult hip fracture if this is clinically and logistically possible.

Regarding mortality of the two groups, there were no deaths in the (small) cohort of patients with a confirmed fracture. Given the expected 6.7% 30-day mortality of hip fracture patients, approximately two deaths would be expected in this group. It may be that patients with non-displaced fractures have a lower mortality, but it is equally possible that the low mortality for patients with a fracture in this study was due to chance. Given that this study only looked at inpatient mortality, there may have been deaths following discharge that are not accounted for. The 3.3% mortality within the non-fracture cohort is lower than that expected following fracture, but still reflects the perilous situation of these patients.

From this evidence, it is our opinion that patients who have a clinically possible but radiographically unproven hip fracture should be initially admitted under a medical speciality used to managing patients with complex medical needs. From an additional medical diagnosis perspective, this paper has shown that this to be the correct admission pathway 73% of the time.

Filling high turnover trauma beds with non-trauma patients who have complex medical, nursing, and social care needs is not an efficient use of a finite acute resource. It places additional pressure on the acute inpatient service with the follow-on effect of longer waiting times for emergent surgical procedures. It also increases the likelihood that patients with a hip fracture, whom we know are at increased risk of mortality, are unable to occupy the appropriate beds in the hip fracture unit and are instead cared for on general medical or surgical wards.

When patients present to the emergency department with a history and examination findings suspicious for hip fracture, but with negative or equivalent plain radiographs, they should be referred to the orthopaedic team for an opinion, and where necessary, cross-sectional imaging should be arranged. If this shows a hip fracture, then it is at this point that orthopaedic surgeons should assume shared care with an orthogeriatric physician. This pathway ensures that patients are managed by the most appropriate team given that the majority of these patients do not require orthopaedic surgery. It is vital that individual hospitals have clearly written guidelines regarding the admission of these patients, to ensure that this advice is followed and to reduce the potential for intra-departmental conflict.

Our aim with this article is not to further increase workloads unnecessarily, but to provide some objective evidence to aid the decision-making process regarding the admission pathway of these patients, thus improving patient care and hospital efficiency.

## Figures and Tables

**Table 1:** Distribution of second-line imaging positive and negative for hip fractures, and associated demographic distributions.

	All	Hip Fracture	No Hip Fracture
*n*	126	34	92
Male	32 (25%)	8 (24%)	24 (26%)
Female	94 (75%)	26 (76%)	68 (74%)
Mean age (range)	83 (66–98)	83 (67–96)	83 (66–98)

**Table 2:** Medical discharge diagnoses of patients with a suspected occult hip fracture subsequently proven to not have a hip fracture.

Diagnosis	*n*	Diagnosis	*n*
Urinary tract infection/urosepsis	14	Monoclonal gammopathy of unknown significance	1
Hospital-acquired pneumonia	9	Constipation	1
Community acquired pneumonia	7	Self-neglect	1
Acute kidney injury	5	Metastatic cancer of unknown origin	1
Delirium	4	Advanced dementia	1
Heart failure (all subtypes)	4	Hallucinations due to Parkinson’s disease	1
Hyponatraemia	2	Hypoglycaemia	1
Multifactorial hypoxia	1	Hypokalaemia	1
Infected leg ulcer	1	Urinary retention	1
Intracerebral haemorrhage	1	Autonomic dysfunction	1
Postural hypotension	1	Paroxysmal atrial fibrillation	1
Anaemia of unknown origin	1	Ischaemic stroke	1
Cellulitis	1	Per-vaginal bleed	1
Aspiration pneumonia	1	Paget’s disease of bone	1
Multisystem atrophy	1	Pyloric stenosis	1
Upper gastrointestinal bleed	1		
